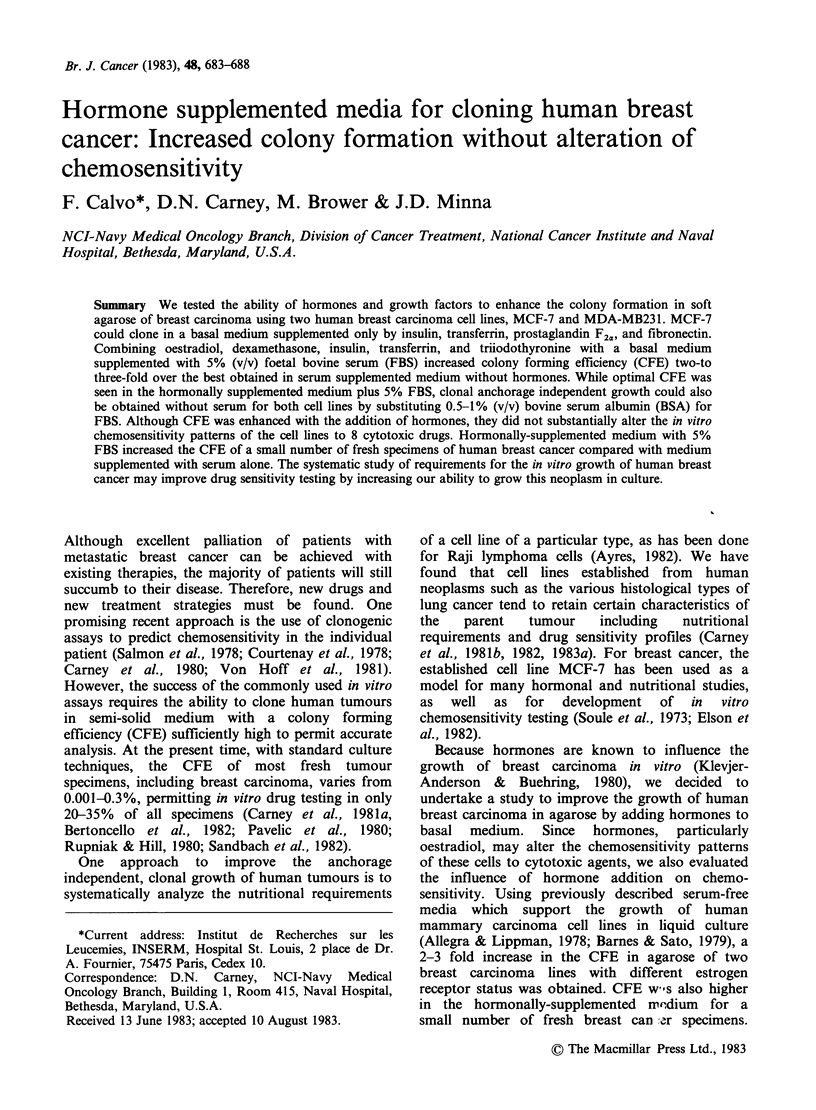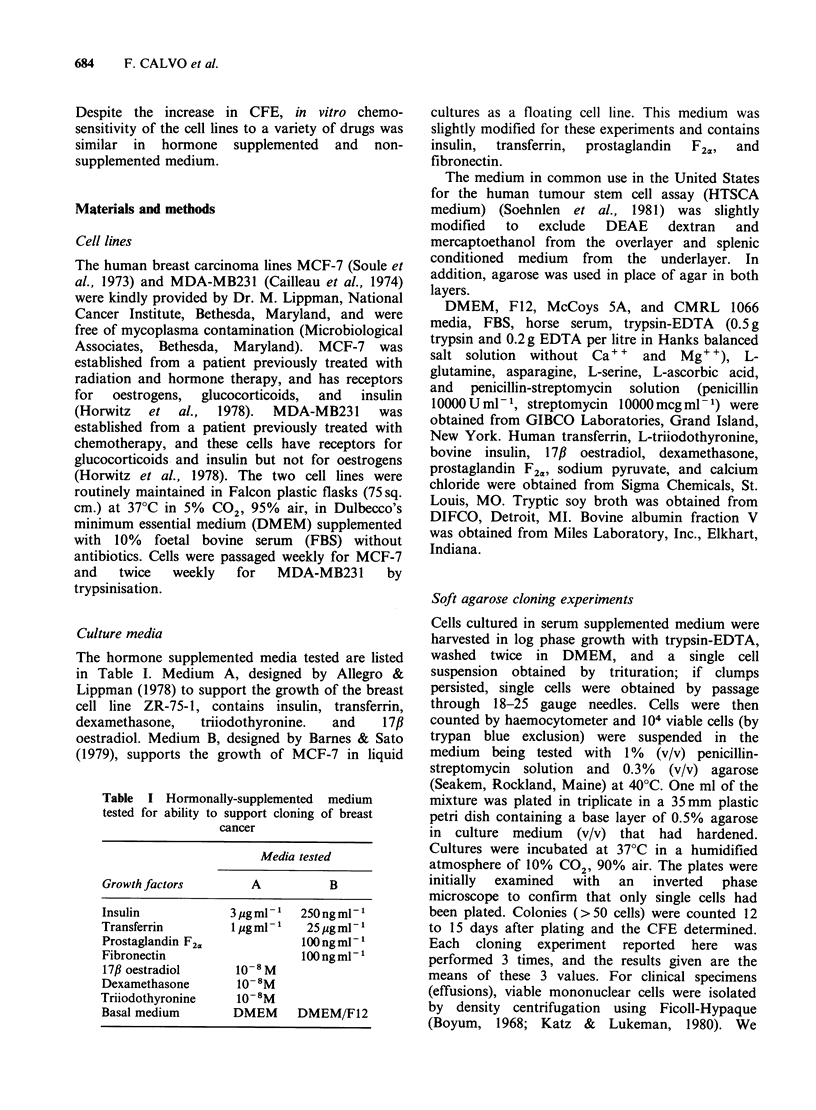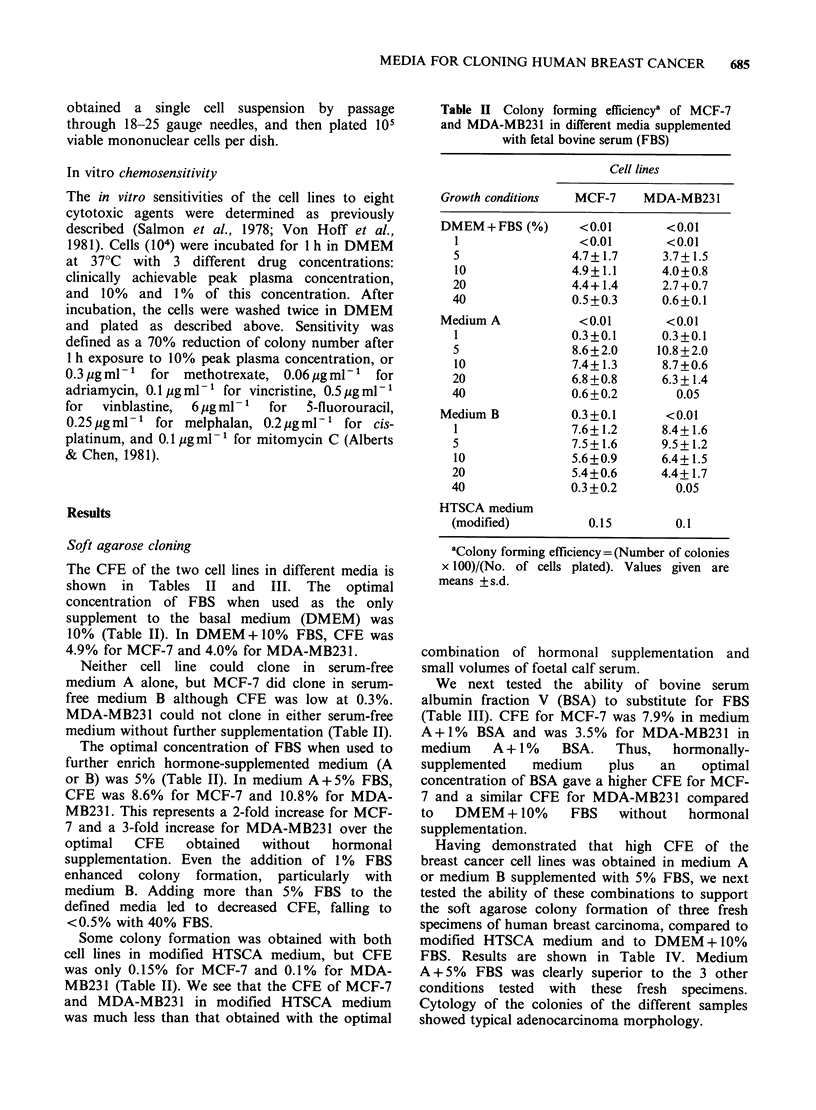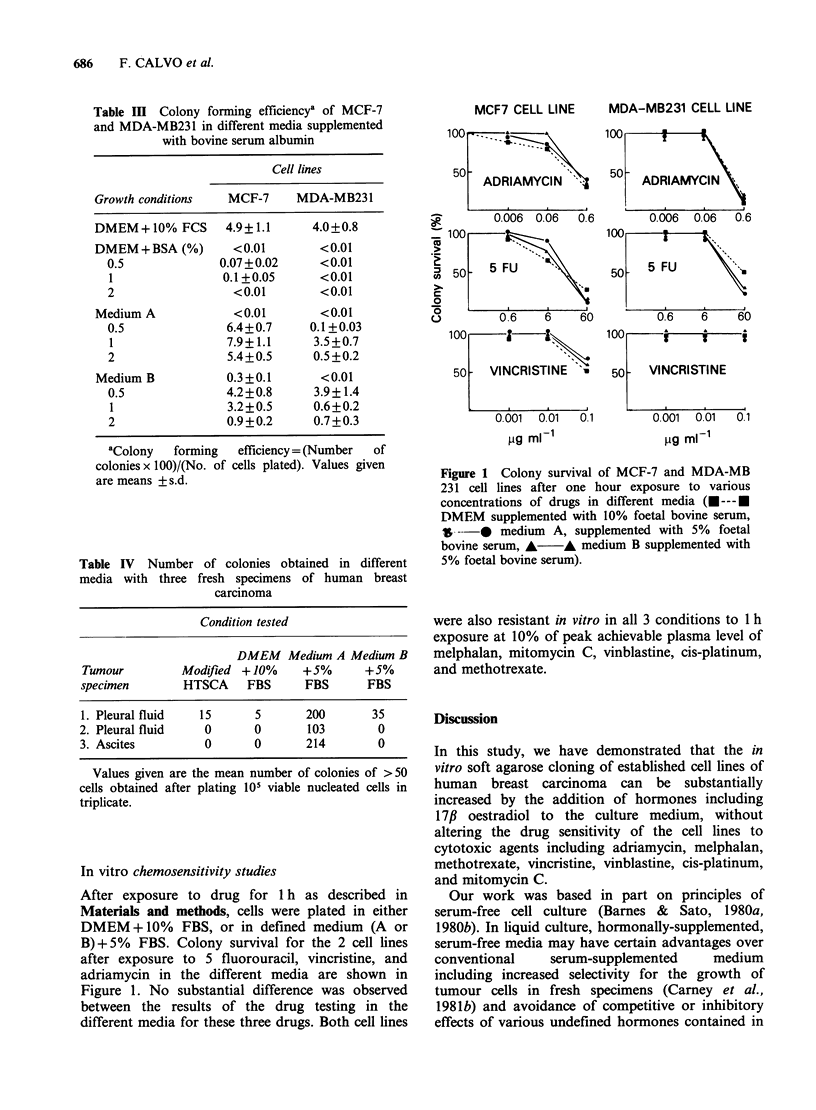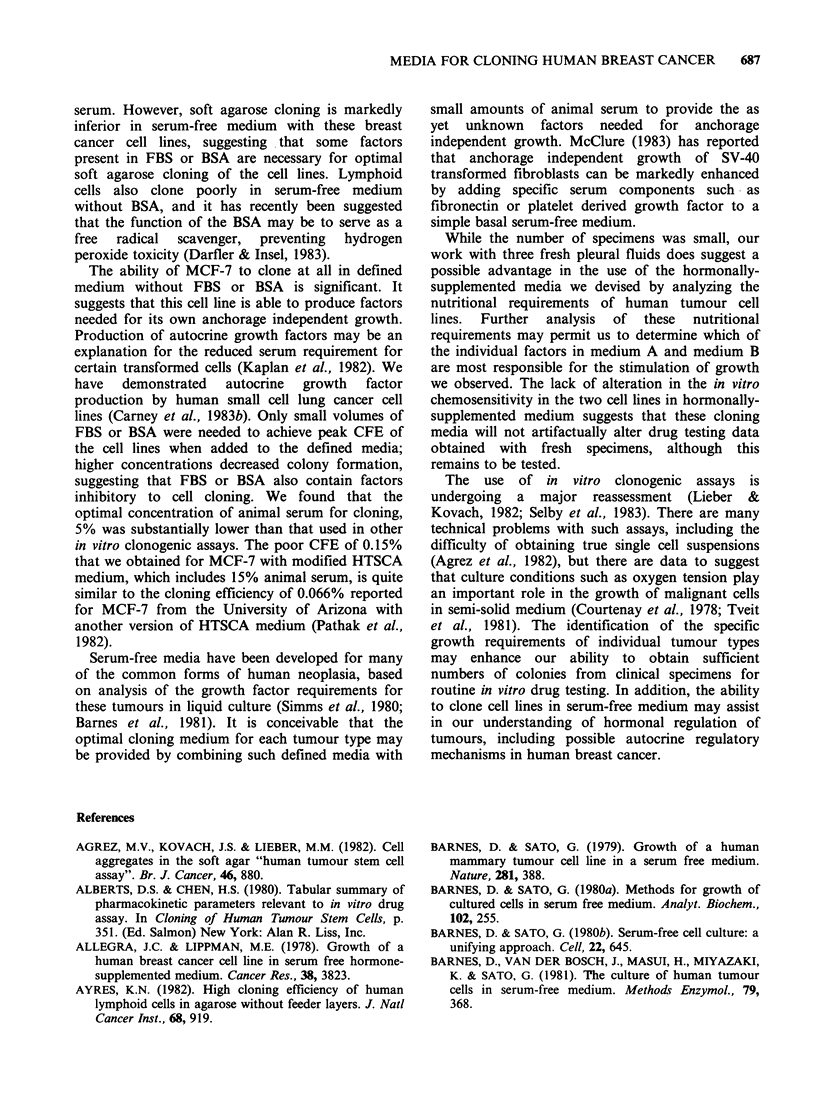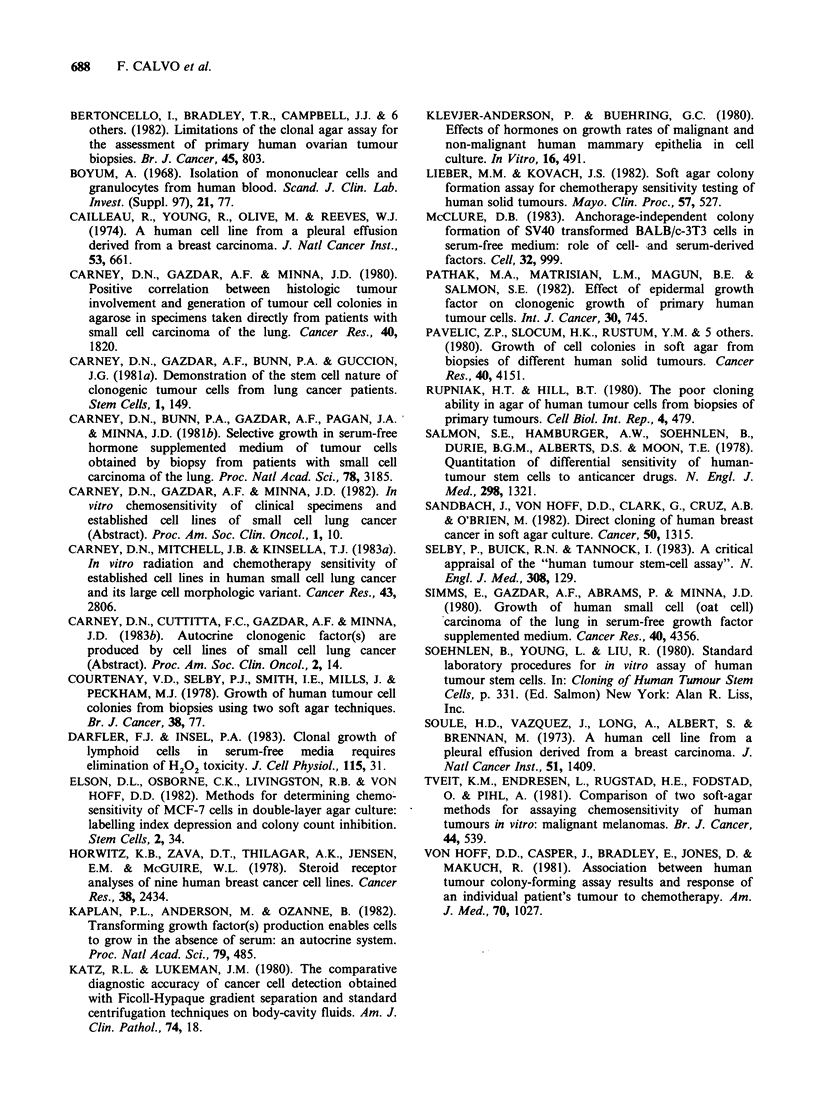# Hormone supplemented media for cloning human breast cancer: increased colony formation without alteration of chemosensitivity.

**DOI:** 10.1038/bjc.1983.250

**Published:** 1983-11

**Authors:** F. Calvo, D. N. Carney, M. Brower, J. D. Minna

## Abstract

We tested the ability of hormones and growth factors to enhance the colony formation in soft agarose of breast carcinoma using two human breast carcinoma cell lines, MCF-7 and MDA-MB231, MCF-7 could clone in a basal medium supplemented only by insulin, transferrin, prostaglandin F2 alpha, and fibronectin. Combining oestradiol, dexamethasone, insulin, transferrin, and triiodothyronine with a basal medium supplemented with 5% (v/v) foetal bovine serum (FBS) increased colony forming efficiency (CFE) two-to three-fold over the best obtained in serum supplemented medium without hormones. While optimal CFE was seen in the hormonally supplemented medium plus 5% FBS, clonal anchorage independent growth could also be obtained without serum for both cell lines by substituting 0.5-1% (v/v) bovine serum albumin (BSA) for FBS. Although CFE was enhanced with the addition of hormones, they did not substantially alter the in vitro chemosensitivity patterns of the cell lines to 8 cytotoxic drugs. Hormonally-supplemented medium with 5% FBS increased the CFE of a small number of fresh specimens of human breast cancer compared with medium supplemented with serum alone. The systematic study of requirements for the in vitro growth of human breast cancer may improve drug sensitivity testing by increasing our ability to grow this neoplasm in culture.


Br. J. Cancer (1983), 48, 683–688

### Hormone supplemented media for cloning human breast cancer: Increased colony formation without alteration of chemosensitivity

F. Calvo*, D.N. Carney, M. Brower & J.D. Minna

*NCI-Navy Medical Oncology Branch, Division of Cancer Treatment, National Cancer Institute and Naval Hospital, Bethesda, Maryland, U.S.A.*

**Summary** We tested the ability of hormones and growth factors to enhance the colony formation in soft agarose of breast carcinoma using two human breast carcinoma cell lines, MCF-7 and MDA-MB231. MCF-7 could clone in a basal medium supplemented only by insulin, transferrin, prostaglandin $F_{2\alpha}$, and fibronectin. Combining oestradiol, dexamethasone, insulin, transferrin, and triiodothyronine with a basal medium supplemented with 5% (v/v) foetal bovine serum (FBS) increased colony forming efficiency (CFE) two-to three-fold over the best obtained in serum supplemented medium without hormones. While optimal CFE was seen in the hormonally supplemented medium plus 5% FBS, clonal anchorage independent growth could also be obtained without serum for both cell lines by substituting 0.5–1% (v/v) bovine serum albumin (BSA) for FBS. Although CFE was enhanced with the addition of hormones, they did not substantially alter the *in vitro* chemosensitivity patterns of the cell lines to 8 cytotoxic drugs. Hormonally-supplemented medium with 5% FBS increased the CFE of a small number of fresh specimens of human breast cancer compared with medium supplemented with serum alone. The systematic study of requirements for the *in vitro* growth of human breast cancer may improve drug sensitivity testing by increasing our ability to grow this neoplasm in culture.

Although excellent palliation of patients with metastatic breast cancer can be achieved with existing therapies, the majority of patients will still succumb to their disease. Therefore, new drugs and new treatment strategies must be found. One promising recent approach is the use of clonogenic assays to predict chemosensitivity in the individual patient (Salmon *et al.*, 1978; Courtenay *et al.*, 1978; Carney *et al.*, 1980; Von Hoff *et al.*, 1981). However, the success of the commonly used *in vitro* assays requires the ability to clone human tumours in semi-solid medium with a colony forming efficiency (CFE) sufficiently high to permit accurate analysis. At the present time, with standard culture techniques, the CFE of most fresh tumour specimens, including breast carcinoma, varies from 0.001–0.3%, permitting *in vitro* drug testing in only 20–35% of all specimens (Carney *et al.*, 1981*a*, Bertoncello *et al.*, 1982; Pavelic *et al.*, 1980; Rupniak & Hill, 1980; Sandbach *et al.*, 1982).

One approach to improve the anchorage independent, clonal growth of human tumours is to systematically analyze the nutritional requirements of a cell line of a particular type, as has been done for Raji lymphoma cells (Ayres, 1982). We have found that cell lines established from human neoplasms such as the various histological types of lung cancer tend to retain certain characteristics of the parent tumour including nutritional requirements and drug sensitivity profiles (Carney *et al.*, 1981*b*, 1982, 1983*a*). For breast cancer, the established cell line MCF-7 has been used as a model for many hormonal and nutritional studies, as well as for development of *in vitro* chemosensitivity testing (Soule *et al.*, 1973; Elson *et al.*, 1982).

Because hormones are known to influence the growth of breast carcinoma *in vitro* (Klevjer-Anderson & Buehring, 1980), we decided to undertake a study to improve the growth of human breast carcinoma in agarose by adding hormones to basal medium. Since hormones, particularly oestradiol, may alter the chemosensitivity patterns of these cells to cytotoxic agents, we also evaluated the influence of hormone addition on chemosensitivity. Using previously described serum-free media which support the growth of human mammary carcinoma cell lines in liquid culture (Allegra & Lippman, 1978; Barnes & Sato, 1979), a 2–3 fold increase in the CFE in agarose of two breast carcinoma lines with different estrogen receptor status was obtained. CFE w·s also higher in the hormonally-supplemented m·dium for a small number of fresh breast can·er specimens.

*Current address: Institut de Recherches sur les Leucemies, INSERM, Hospital St. Louis, 2 place de Dr. A. Fournier, 75475 Paris, Cedex 10.
Correspondence: D.N. Carney, NCI-Navy Medical Oncology Branch, Building 1, Room 415, Naval Hospital, Bethesda, Maryland, U.S.A.
Received 13 June 1983; accepted 10 August 1983.

© The Macmillar Press Ltd., 1983

Despite the increase in CFE, *in vitro* chemosensitivity of the cell lines to a variety of drugs was similar in hormone supplemented and non-supplemented medium.

### Materials and methods

### *Cell lines*

The human breast carcinoma lines MCF-7 (Soule *et al.*, 1973) and MDA-MB231 (Cailleau *et al.*, 1974) were kindly provided by Dr. M. Lippman, National Cancer Institute, Bethesda, Maryland, and were free of mycoplasma contamination (Microbiological Associates, Bethesda, Maryland). MCF-7 was established from a patient previously treated with radiation and hormone therapy, and has receptors for oestrogens, glucocorticoids, and insulin (Horwitz *et al.*, 1978). MDA-MB231 was established from a patient previously treated with chemotherapy, and these cells have receptors for glucocorticoids and insulin but not for oestrogens (Horwitz *et al.*, 1978). The two cell lines were routinely maintained in Falcon plastic flasks (75 sq. cm.) at 37°C in 5% $CO_2$, 95% air, in Dulbecco's minimum essential medium (DMEM) supplemented with 10% foetal bovine serum (FBS) without antibiotics. Cells were passaged weekly for MCF-7 and twice weekly for MDA-MB231 by trypsinisation.

### *Culture media*

The hormone supplemented media tested are listed in Table I. Medium A, designed by Allegro & Lippman (1978) to support the growth of the breast cell line ZR-75-1, contains insulin, transferrin, dexamethasone, triiodothyronine. and 17$\beta$ oestradiol. Medium B, designed by Barnes & Sato (1979), supports the growth of MCF-7 in liquid cultures as a floating cell line. This medium was slightly modified for these experiments and contains insulin, transferrin, prostaglandin $F_{2\alpha}$, and fibronectin.

**Table I** Hormonally-supplemented medium tested for ability to support cloning of breast cancer

| | *Media tested* | |
|---|---|---|
| *Growth factors* | A | B |
| Insulin | 3 $\mu g\,ml^{-1}$ | 250 $ng\,ml^{-1}$ |
| Transferrin | 1 $\mu g\,ml^{-1}$ | 25 $\mu g\,ml^{-1}$ |
| Prostaglandin $F_{2\alpha}$ | | 100 $ng\,ml^{-1}$ |
| Fibronectin | | 100 $ng\,ml^{-1}$ |
| 17$\beta$ oestradiol | $10^{-8}$ M | |
| Dexamethasone | $10^{-8}$ M | |
| Triiodothyronine | $10^{-8}$ M | |
| Basal medium | DMEM | DMEM/F12 |

The medium in common use in the United States for the human tumour stem cell assay (HTSCA medium) (Soehnlen *et al.*, 1981) was slightly modified to exclude DEAE dextran and mercaptoethanol from the overlayer and splenic conditioned medium from the underlayer. In addition, agarose was used in place of agar in both layers.

DMEM, F12, McCoys 5A, and CMRL 1066 media, FBS, horse serum, trypsin-EDTA (0.5 g trypsin and 0.2 g EDTA per litre in Hanks balanced salt solution without $Ca^{++}$ and $Mg^{++}$), L-glutamine, asparagine, L-serine, L-ascorbic acid, and penicillin-streptomycin solution (penicillin 10000 $U\,ml^{-1}$, streptomycin 10000 $mcg\,ml^{-1}$) were obtained from GIBCO Laboratories, Grand Island, New York. Human transferrin, L-triiodothyronine, bovine insulin, 17$\beta$ oestradiol, dexamethasone, prostaglandin $F_{2\alpha}$, sodium pyruvate, and calcium chloride were obtained from Sigma Chemicals, St. Louis, MO. Tryptic soy broth was obtained from DIFCO, Detroit, MI. Bovine albumin fraction V was obtained from Miles Laboratory, Inc., Elkhart, Indiana.

### *Soft agarose cloning experiments*

Cells cultured in serum supplemented medium were harvested in log phase growth with trypsin-EDTA, washed twice in DMEM, and a single cell suspension obtained by trituration; if clumps persisted, single cells were obtained by passage through 18–25 gauge needles. Cells were then counted by haemocytometer and $10^4$ viable cells (by trypan blue exclusion) were suspended in the medium being tested with 1% (v/v) penicillin-streptomycin solution and 0.3% (v/v) agarose (Seakem, Rockland, Maine) at 40°C. One ml of the mixture was plated in triplicate in a 35 mm plastic petri dish containing a base layer of 0.5% agarose in culture medium (v/v) that had hardened. Cultures were incubated at 37°C in a humidified atmosphere of 10% $CO_2$, 90% air. The plates were initially examined with an inverted phase microscope to confirm that only single cells had been plated. Colonies (>50 cells) were counted 12 to 15 days after plating and the CFE determined. Each cloning experiment reported here was performed 3 times, and the results given are the means of these 3 values. For clinical specimens (effusions), viable mononuclear cells were isolated by density centrifugation using Ficoll-Hypaque (Boyum, 1968; Katz & Lukeman, 1980). We obtained a single cell suspension by passage through 18–25 gauge needles, and then plated $10^5$ viable mononuclear cells per dish.

### *In vitro chemosensitivity*

The *in vitro* sensitivities of the cell lines to eight cytotoxic agents were determined as previously described (Salmon *et al.*, 1978; Von Hoff *et al.*, 1981). Cells ($10^4$) were incubated for 1 h in DMEM at 37°C with 3 different drug concentrations: clinically achievable peak plasma concentration, and 10% and 1% of this concentration. After incubation, the cells were washed twice in DMEM and plated as described above. Sensitivity was defined as a 70% reduction of colony number after 1 h exposure to 10% peak plasma concentration, or 0.3 $\mu g\,ml^{-1}$ for methotrexate, 0.06 $\mu g\,ml^{-1}$ for adriamycin, 0.1 $\mu g\,ml^{-1}$ for vincristine, 0.5 $\mu g\,ml^{-1}$ for vinblastine, 6 $\mu g\,ml^{-1}$ for 5-fluorouracil, 0.25 $\mu g\,ml^{-1}$ for melphalan, 0.2 $\mu g\,ml^{-1}$ for *cis*-platinum, and 0.1 $\mu g\,ml^{-1}$ for mitomycin C (Alberts & Chen, 1981).

### Results

### *Soft agarose cloning*

The CFE of the two cell lines in different media is shown in Tables II and III. The optimal concentration of FBS when used as the only supplement to the basal medium (DMEM) was 10% (Table II). In DMEM + 10% FBS, CFE was 4.9% for MCF-7 and 4.0% for MDA-MB231.

Neither cell line could clone in serum-free medium A alone, but MCF-7 did clone in serum-free medium B although CFE was low at 0.3%. MDA-MB231 could not clone in either serum-free medium without further supplementation (Table II).

The optimal concentration of FBS when used to further enrich hormone-supplemented medium (A or B) was 5% (Table II). In medium A + 5% FBS, CFE was 8.6% for MCF-7 and 10.8% for MDA-MB231. This represents a 2-fold increase for MCF-7 and a 3-fold increase for MDA-MB231 over the optimal CFE obtained without hormonal supplementation. Even the addition of 1% FBS enhanced colony formation, particularly with medium B. Adding more than 5% FBS to the defined media led to decreased CFE, falling to <0.5% with 40% FBS.

Some colony formation was obtained with both cell lines in modified HTSCA medium, but CFE was only 0.15% for MCF-7 and 0.1% for MDA-MB231 (Table II). We see that the CFE of MCF-7 and MDA-MB231 in modified HTSCA medium was much less than that obtained with the optimal combination of hormonal supplementation and small volumes of foetal calf serum.

**Table II** Colony forming efficiency[a] of MCF-7 and MDA-MB231 in different media supplemented with fetal bovine serum (FBS)

| | Cell lines | |
|---|---|---|
| *Growth conditions* | MCF-7 | MDA-MB231 |
| DMEM + FBS (%) | <0.01 | <0.01 |
| 1 | <0.01 | <0.01 |
| 5 | 4.7 ± 1.7 | 3.7 ± 1.5 |
| 10 | 4.9 ± 1.1 | 4.0 ± 0.8 |
| 20 | 4.4 + 1.4 | 2.7 + 0.7 |
| 40 | 0.5 ± 0.3 | 0.6 ± 0.1 |
| Medium A | <0.01 | <0.01 |
| 1 | 0.3 ± 0.1 | 0.3 ± 0.1 |
| 5 | 8.6 ± 2.0 | 10.8 ± 2.0 |
| 10 | 7.4 ± 1.3 | 8.7 ± 0.6 |
| 20 | 6.8 ± 0.8 | 6.3 ± 1.4 |
| 40 | 0.6 ± 0.2 | 0.05 |
| Medium B | 0.3 ± 0.1 | <0.01 |
| 1 | 7.6 ± 1.2 | 8.4 ± 1.6 |
| 5 | 7.5 ± 1.6 | 9.5 ± 1.2 |
| 10 | 5.6 ± 0.9 | 6.4 ± 1.5 |
| 20 | 5.4 ± 0.6 | 4.4 ± 1.7 |
| 40 | 0.3 ± 0.2 | 0.05 |
| HTSCA medium (modified) | 0.15 | 0.1 |

[a]Colony forming efficiency = (Number of colonies × 100)/(No. of cells plated). Values given are means ± s.d.

We next tested the ability of bovine serum albumin fraction V (BSA) to substitute for FBS (Table III). CFE for MCF-7 was 7.9% in medium A + 1% BSA and was 3.5% for MDA-MB231 in medium A + 1% BSA. Thus, hormonally-supplemented medium plus an optimal concentration of BSA gave a higher CFE for MCF-7 and a similar CFE for MDA-MB231 compared to DMEM + 10% FBS without hormonal supplementation.

Having demonstrated that high CFE of the breast cancer cell lines was obtained in medium A or medium B supplemented with 5% FBS, we next tested the ability of these combinations to support the soft agarose colony formation of three fresh specimens of human breast carcinoma, compared to modified HTSCA medium and to DMEM + 10% FBS. Results are shown in Table IV. Medium A + 5% FBS was clearly superior to the 3 other conditions tested with these fresh specimens. Cytology of the colonies of the different samples showed typical adenocarcinoma morphology.

**Table III** Colony forming efficiency[a] of MCF-7 and MDA-MB231 in different media supplemented with bovine serum albumin

| Growth conditions | Cell lines MCF-7 | MDA-MB231 |
|---|---|---|
| DMEM+10% FCS | 4.9±1.1 | 4.0±0.8 |
| DMEM+BSA (%) | <0.01 | <0.01 |
| 0.5 | 0.07±0.02 | <0.01 |
| 1 | 0.1±0.05 | <0.01 |
| 2 | <0.01 | <0.01 |
| Medium A | <0.01 | <0.01 |
| 0.5 | 6.4±0.7 | 0.1±0.03 |
| 1 | 7.9±1.1 | 3.5±0.7 |
| 2 | 5.4±0.5 | 0.5±0.2 |
| Medium B | 0.3±0.1 | <0.01 |
| 0.5 | 4.2±0.8 | 3.9±1.4 |
| 1 | 3.2±0.5 | 0.6±0.2 |
| 2 | 0.9±0.2 | 0.7±0.3 |

[a]Colony forming efficiency=(Number of colonies × 100)/(No. of cells plated). Values given are means ±s.d.

**Table IV** Number of colonies obtained in different media with three fresh specimens of human breast carcinoma

| Tumour specimen | Condition tested: Modified HTSCA | DMEM +10% FBS | Medium A +5% FBS | Medium B +5% FBS |
|---|---|---|---|---|
| 1. Pleural fluid | 15 | 5 | 200 | 35 |
| 2. Pleural fluid | 0 | 0 | 103 | 0 |
| 3. Ascites | 0 | 0 | 214 | 0 |

Values given are the mean number of colonies of >50 cells obtained after plating $10^5$ viable nucleated cells in triplicate.

**Figure 1** Colony survival of MCF-7 and MDA-MB 231 cell lines after one hour exposure to various concentrations of drugs in different media (■---■ DMEM supplemented with 10% foetal bovine serum, ●——● medium A, supplemented with 5% foetal bovine serum, ▲——▲ medium B supplemented with 5% foetal bovine serum).

### In vitro *chemosensitivity studies*

After exposure to drug for 1 h as described in **Materials and methods**, cells were plated in either DMEM+10% FBS, or in defined medium (A or B)+5% FBS. Colony survival for the 2 cell lines after exposure to 5 fluorouracil, vincristine, and adriamycin in the different media are shown in Figure 1. No substantial difference was observed between the results of the drug testing in the different media for these three drugs. Both cell lines were also resistant *in vitro* in all 3 conditions to 1 h exposure at 10% of peak achievable plasma level of melphalan, mitomycin C, vinblastine, cis-platinum, and methotrexate.

### Discussion

In this study, we have demonstrated that the *in vitro* soft agarose cloning of established cell lines of human breast carcinoma can be substantially increased by the addition of hormones including 17β oestradiol to the culture medium, without altering the drug sensitivity of the cell lines to cytotoxic agents including adriamycin, melphalan, methotrexate, vincristine, vinblastine, cis-platinum, and mitomycin C.

Our work was based in part on principles of serum-free cell culture (Barnes & Sato, 1980*a*, 1980*b*). In liquid culture, hormonally-supplemented, serum-free media may have certain advantages over conventional serum-supplemented medium including increased selectivity for the growth of tumour cells in fresh specimens (Carney *et al.*, 1981*b*) and avoidance of competitive or inhibitory effects of various undefined hormones contained in serum. However, soft agarose cloning is markedly inferior in serum-free medium with these breast cancer cell lines, suggesting that some factors present in FBS or BSA are necessary for optimal soft agarose cloning of the cell lines. Lymphoid cells also clone poorly in serum-free medium without BSA, and it has recently been suggested that the function of the BSA may be to serve as a free radical scavenger, preventing hydrogen peroxide toxicity (Darfler & Insel, 1983).

The ability of MCF-7 to clone at all in defined medium without FBS or BSA is significant. It suggests that this cell line is able to produce factors needed for its own anchorage independent growth. Production of autocrine growth factors may be an explanation for the reduced serum requirement for certain transformed cells (Kaplan *et al.*, 1982). We have demonstrated autocrine growth factor production by human small cell lung cancer cell lines (Carney *et al.*, 1983*b*). Only small volumes of FBS or BSA were needed to achieve peak CFE of the cell lines when added to the defined media; higher concentrations decreased colony formation, suggesting that FBS or BSA also contain factors inhibitory to cell cloning. We found that the optimal concentration of animal serum for cloning, 5% was substantially lower than that used in other *in vitro* clonogenic assays. The poor CFE of 0.15% that we obtained for MCF-7 with modified HTSCA medium, which includes 15% animal serum, is quite similar to the cloning efficiency of 0.066% reported for MCF-7 from the University of Arizona with another version of HTSCA medium (Pathak *et al.*, 1982).

Serum-free media have been developed for many of the common forms of human neoplasia, based on analysis of the growth factor requirements for these tumours in liquid culture (Simms *et al.*, 1980; Barnes *et al.*, 1981). It is conceivable that the optimal cloning medium for each tumour type may be provided by combining such defined media with small amounts of animal serum to provide the as yet unknown factors needed for anchorage independent growth. McClure (1983) has reported that anchorage independent growth of SV-40 transformed fibroblasts can be markedly enhanced by adding specific serum components such as fibronectin or platelet derived growth factor to a simple basal serum-free medium.

While the number of specimens was small, our work with three fresh pleural fluids does suggest a possible advantage in the use of the hormonally-supplemented media we devised by analyzing the nutritional requirements of human tumour cell lines. Further analysis of these nutritional requirements may permit us to determine which of the individual factors in medium A and medium B are most responsible for the stimulation of growth we observed. The lack of alteration in the *in vitro* chemosensitivity in the two cell lines in hormonally-supplemented medium suggests that these cloning media will not artifactually alter drug testing data obtained with fresh specimens, although this remains to be tested.

The use of *in vitro* clonogenic assays is undergoing a major reassessment (Lieber & Kovach, 1982; Selby *et al.*, 1983). There are many technical problems with such assays, including the difficulty of obtaining true single cell suspensions (Agrez *et al.*, 1982), but there are data to suggest that culture conditions such as oxygen tension play an important role in the growth of malignant cells in semi-solid medium (Courtenay *et al.*, 1978; Tveit *et al.*, 1981). The identification of the specific growth requirements of individual tumour types may enhance our ability to obtain sufficient numbers of colonies from clinical specimens for routine *in vitro* drug testing. In addition, the ability to clone cell lines in serum-free medium may assist in our understanding of hormonal regulation of tumours, including possible autocrine regulatory mechanisms in human breast cancer.

### References

AGREZ, M.V., KOVACH, J.S. & LIEBER, M.M. (1982). Cell aggregates in the soft agar "human tumour stem cell assay". *Br. J. Cancer*, **46**, 880.

ALBERTS, D.S. & CHEN, H.S. (1980). Tabular summary of pharmacokinetic parameters relevant to *in vitro* drug assay. In *Cloning of Human Tumour Stem Cells*, p. 351. (Ed. Salmon) New York: Alan R. Liss, Inc.

ALLEGRA, J.C. & LIPPMAN, M.E. (1978). Growth of a human breast cancer cell line in serum free hormone-supplemented medium. *Cancer Res.*, **38**, 3823.

AYRES, K.N. (1982). High cloning efficiency of human lymphoid cells in agarose without feeder layers. *J. Natl Cancer Inst.*, **68**, 919.

BARNES, D. & SATO, G. (1979). Growth of a human mammary tumour cell line in a serum free medium. *Nature*, **281**, 388.

BARNES, D. & SATO, G. (1980*a*). Methods for growth of cultured cells in serum free medium. *Analyt. Biochem.*, **102**, 255.

BARNES, D. & SATO, G. (1980*b*). Serum-free cell culture: a unifying approach. *Cell*, **22**, 645.

BARNES, D., VAN DER BOSCH, J., MASUI, H., MIYAZAKI, K. & SATO, G. (1981). The culture of human tumour cells in serum-free medium. *Methods Enzymol.*, **79**, 368.

BERTONCELLO, I., BRADLEY, T.R., CAMPBELL, J.J. & 6 others. (1982). Limitations of the clonal agar assay for the assessment of primary human ovarian tumour biopsies. *Br. J. Cancer*, **45**, 803.

BOYUM, A. (1968). Isolation of mononuclear cells and granulocytes from human blood. *Scand. J. Clin. Lab. Invest.* (Suppl. 97), **21**, 77.

CAILLEAU, R., YOUNG, R., OLIVE, M. & REEVES, W.J. (1974). A human cell line from a pleural effusion derived from a breast carcinoma. *J. Natl Cancer Inst.*, **53**, 661.

CARNEY, D.N., GAZDAR, A.F. & MINNA, J.D. (1980). Positive correlation between histologic tumour involvement and generation of tumour cell colonies in agarose in specimens taken directly from patients with small cell carcinoma of the lung. *Cancer Res.*, **40**, 1820.

CARNEY, D.N., GAZDAR, A.F., BUNN, P.A. & GUCCION, J.G. (1981*a*). Demonstration of the stem cell nature of clonogenic tumour cells from lung cancer patients. *Stem Cells*, **1**, 149.

CARNEY, D.N., BUNN, P.A., GAZDAR, A.F., PAGAN, J.A. & MINNA, J.D. (1981*b*). Selective growth in serum-free hormone supplemented medium of tumour cells obtained by biopsy from patients with small cell carcinoma of the lung. *Proc. Natl Acad. Sci.*, **78**, 3185.

CARNEY, D.N., GAZDAR, A.F. & MINNA, J.D. (1982). *In vitro* chemosensitivity of clinical specimens and established cell lines of small cell lung cancer (Abstract). *Proc. Am. Soc. Clin. Oncol.*, **1**, 10.

CARNEY, D.N., MITCHELL, J.B. & KINSELLA, T.J. (1983*a*). *In vitro* radiation and chemotherapy sensitivity of established cell lines in human small cell lung cancer and its large cell morphologic variant. *Cancer Res.*, **43**, 2806.

CARNEY, D.N., CUTTITTA, F.C., GAZDAR, A.F. & MINNA, J.D. (1983*b*). Autocrine clonogenic factor(s) are produced by cell lines of small cell lung cancer (Abstract). *Proc. Am. Soc. Clin. Oncol.*, **2**, 14.

COURTENAY, V.D., SELBY, P.J., SMITH, I.E., MILLS, J. & PECKHAM, M.J. (1978). Growth of human tumour cell colonies from biopsies using two soft agar techniques. *Br. J. Cancer*, **38**, 77.

DARFLER, F.J. & INSEL, P.A. (1983). Clonal growth of lymphoid cells in serum-free media requires elimination of $H_2O_2$ toxicity. *J. Cell Physiol.*, **115**, 31.

ELSON, D.L., OSBORNE, C.K., LIVINGSTON, R.B. & VON HOFF, D.D. (1982). Methods for determining chemosensitivity of MCF-7 cells in double-layer agar culture: labelling index depression and colony count inhibition. *Stem Cells*, **2**, 34.

HORWITZ, K.B., ZAVA, D.T., THILAGAR, A.K., JENSEN, E.M. & McGUIRE, W.L. (1978). Steroid receptor analyses of nine human breast cancer cell lines. *Cancer Res.*, **38**, 2434.

KAPLAN, P.L., ANDERSON, M. & OZANNE, B. (1982). Transforming growth factor(s) production enables cells to grow in the absence of serum: an autocrine system. *Proc. Natl Acad. Sci.*, **79**, 485.

KATZ, R.L. & LUKEMAN, J.M. (1980). The comparative diagnostic accuracy of cancer cell detection obtained with Ficoll-Hypaque gradient separation and standard centrifugation techniques on body-cavity fluids. *Am. J. Clin. Pathol.*, **74**, 18.

KLEVJER-ANDERSON, P. & BUEHRING, G.C. (1980). Effects of hormones on growth rates of malignant and non-malignant human mammary epithelia in cell culture. *In Vitro*, **16**, 491.

LIEBER, M.M. & KOVACH, J.S. (1982). Soft agar colony formation assay for chemotherapy sensitivity testing of human solid tumours. *Mayo. Clin. Proc.*, **57**, 527.

McCLURE, D.B. (1983). Anchorage-independent colony formation of SV40 transformed BALB/c-3T3 cells in serum-free medium: role of cell- and serum-derived factors. *Cell*, **32**, 999.

PATHAK, M.A., MATRISIAN, L.M., MAGUN, B.E. & SALMON, S.E. (1982). Effect of epidermal growth factor on clonogenic growth of primary human tumour cells. *Int. J. Cancer*, **30**, 745.

PAVELIC, Z.P., SLOCUM, H.K., RUSTUM, Y.M. & 5 others. (1980). Growth of cell colonies in soft agar from biopsies of different human solid tumours. *Cancer Res.*, **40**, 4151.

RUPNIAK, H.T. & HILL, B.T. (1980). The poor cloning ability in agar of human tumour cells from biopsies of primary tumours. *Cell Biol. Int. Rep.*, **4**, 479.

SALMON, S.E., HAMBURGER, A.W., SOEHNLEN, B., DURIE, B.G.M., ALBERTS, D.S. & MOON, T.E. (1978). Quantitation of differential sensitivity of human-tumour stem cells to anticancer drugs. *N. Engl. J. Med.*, **298**, 1321.

SANDBACH, J., VON HOFF, D.D., CLARK, G., CRUZ, A.B. & O'BRIEN, M. (1982). Direct cloning of human breast cancer in soft agar culture. *Cancer*, **50**, 1315.

SELBY, P., BUICK, R.N. & TANNOCK, I. (1983). A critical appraisal of the "human tumour stem-cell assay". *N. Engl. J. Med.*, **308**, 129.

SIMMS, E., GAZDAR, A.F., ABRAMS, P. & MINNA, J.D. (1980). Growth of human small cell (oat cell) carcinoma of the lung in serum-free growth factor supplemented medium. *Cancer Res.*, **40**, 4356.

SOEHNLEN, B., YOUNG, L. & LIU, R. (1980). Standard laboratory procedures for *in vitro* assay of human tumour stem cells. In: *Cloning of Human Tumour Stem Cells*, p. 331. (Ed. Salmon) New York: Alan R. Liss, Inc.

SOULE, H.D., VAZQUEZ, J., LONG, A., ALBERT, S. & BRENNAN, M. (1973). A human cell line from a pleural effusion derived from a breast carcinoma. *J. Natl Cancer Inst.*, **51**, 1409.

TVEIT, K.M., ENDRESEN, L., RUGSTAD, H.E., FODSTAD, O. & PIHL, A. (1981). Comparison of two soft-agar methods for assaying chemosensitivity of human tumours *in vitro*: malignant melanomas. *Br. J. Cancer*, **44**, 539.

VON HOFF, D.D., CASPER, J., BRADLEY, E., JONES, D. & MAKUCH, R. (1981). Association between human tumour colony-forming assay results and response of an individual patient's tumour to chemotherapy. *Am. J. Med.*, **70**, 1027.